# The effect of Ankaferd blood stopper on liver damage in experimental obstructive jaundice

**DOI:** 10.3906/sag-2007-298

**Published:** 2021-06-28

**Authors:** Koray KOŞMAZ, Abdullah DURHAN, Marlen SÜLEYMAN, Yılmaz ÜNAL, Mustafa Taner BOSTANCI, Tuğba YİĞİT HASKARACA, Can ERSAK, Mehmet ŞENEŞ, İlknur Alkan KUŞABBİ, Eylem Pınar ESER, Sema HÜCÜMENOĞLU

**Affiliations:** 1 Department of General Surgery, Ankara Education and Research Hospital, Ankara Turkey; 2 Department of General Surgery, Dışkapı Education and Research Hospital, Ankara Turkey; 3 Department of Biochemistry, Ankara Education and Research Hospital, Ankara Turkey; 4 Department of Pathology, Ankara Education and Research Hospital, Ankara Turkey

**Keywords:** Obstructive jaundice, Ankaferd blood stopper, antioxidant

## Abstract

**Background/aims:**

To evaluate the potential protective effects of Ankaferd blood stopper (ABS) in an experimental obstructive jaundice (OJ) model.

**Materials and methods:**

The study included 26 female rats, which were divided into 3 groups. The sham group, consisting of 10 rats, (group 1) only received solely laparotomy. In the control group, consisting of 8 rats, (group 2), ligation was applied to the biliary tract and no treatment was implemented. In the treatment group, consisting of 8 rats, (group 3), following ligation of biliary tract, 0.5 mL/day ABS was given for 10 days. Liver tissue and blood samples were taken for histopathological and biochemical examination.

**Results:**

Compared to group 2, group 3 had higher aspartate aminotransferase (AST), total oxidant status (TOS) malondialdehyde (MDA), fluorescent oxidant products (FOP), and lower expression of albumin and total antioxidant status (TAS) (P < 0.05). In histopathological analysis, the mean scores of all histopathological parameters (fibrosis, portal inflammation, confluent necrosis, interphase activity, bile duct proliferation) have statistical significance between group 2 and group 3 (P < 005).

**Conclusions:**

ABS has promising results in the treatment of experimental OJ because of its antioxidant and antiinflammatory properties. It may be used in clinical practice after more extensive studies about the effects of ABS on OJ.

## 1. Introduction

Obstructive jaundice (OJ) occurs with blockage of the bile stream in the extrahepatic or intrahepatic biliary tract. This obstruction in the pathway of bile synthesis through the gastrointestinal system causes cholestasis [1].

The hepatotoxic effects of bile acids, oxidative stress and lipid peroxidation are the main issues for liver damage caused by cholestasis [2]. Complications of insufficient treatment are cholangitis, coagulation disorders, biliary fibrosis and hepatic damage leading to cirrhosis [3]. Therefore, various agents and methods have been studied to decrease the damage caused by obstructive jaundice, but these agents are not medications that are widely used in clinical practice [4].

Ankaferd blood stopper (ABS) (Ankaferd Health Products Ltd., İstanbul, Turkey) is a topical haemostatic agent of plant origin that has come into use in recent years. ABS forms an encapsulated protein network that provides critical junction points for rapid erythrocyte aggregation without affecting physiological coagulation systems [5]. In addition to haemostatic functions, antiinflammatory, antiinfective, antifungal, and antioxidative effects have been attributed to ABS [6–8]. This herbal medication contains a standard combination of Thymus vulgaris, Glychrriza glabra, Vitris vinifera, Alpina officinarum, and Urtica dioicayi. Each of these plants has effects on endothelial and blood cells, angiogenesis, cell proliferation, vascular dynamics and interaction between cells. There are some examples of T. vulgaris having an antioxidant effect by inhibiting lipid peroxidation and U. Dionica has been shown to cause vasodilatation by inducing nitric oxide (NO) synthesis from the vascular endothelium. There are limited studies on clinical and experimental usage of ABS in hepatic surgeries [5].

The aim of this experimental study was to investigate the effects of ABS on experimental obstructive jaundice through the evaluation of oxidative stress and pathological changes. To the best of our knowledge, this is the first study in the literature to have examined the effect of ABS on hepatic damage caused by obstructive jaundice.

## 2. Material and methods

The approval for this study was granted by the Animal Research Ethics Committee of Ankara Training and Research Hospital and all procedures were applied in accordance with the principles of the National Guidelines for Experimental Use of Laboratory Animals.

### 2.1. Animals

The study sample consisted of 30 female adult Wistar albino rats, each with a weight of 230 ± 22 g. The rats were housed in cages made of wire netting at a permanent temperature of 21 ± 2 °C with a 12-h light/dark cycle. The animals had a diet consisting of standard laboratory supply chow and water ad libitum. Access to food and access to water for the rats was stopped 12 h and 2 h prior to anaesthesia, respectively.

### 2.2. Surgery and experimental protocol

This research aimed to investigate the effects of Ankaferd blood stopper on obstructive jaundice. The group plans are explained below:

Group 1: Laparotomy was applied, and then the choledochus was dissected from adjacent tissues. No other treatment was given.

Group 2: Laparotomy was applied, and then the choledochus was dissected from adjacent tissue and tied twice with 5/0 silk sutures. The choledochus was cut between the sutures. No other intervention or medication was given.

Group 3: Laparotomy was applied and then the choledochus was dissected from adjacent tissues and tied twice with 5/0 silk sutures. The choledochus was cut between the sutures. Ankaferd blood stopper was administered by orogastric catheter at a dose of 0.5 mL per day.

After the procedures, each abdominal incision was closed with 3/0 silk sutures. Subsequently, the rats were allowed to feed. After 10 days, the rats were slaughtered with high dose ketamine, and then laparotomy was performed. In laparotomy, blood samples were taken from the aorta for histopathological and biochemical analyses, and a 40 mg tissue sample was taken from the liver as excision.

### 2.3. Biochemical analysis

The evaluation of the liver functions in the serum was carried out in the Biochemistry Department of Ankara Training and Research Hospital. Albumin (ALB), aspartate aminotransferase (AST), alanine aminotransferase (ALT), total bilirubin (T. BIL), and direct bilirubin (D. BIL) were measured using a Roche Cobas 8000 chemistry analyser (Roche Diagnostics, Risch-Rotkreuz, Switzerland).

The parameters of oxidative stress were evaluated in the Biochemistry Department of Ankara Training and Research Hospital. Liver tissues were kept at −80 °C until the day of the analysis. Total antioxidant status (TAS), total oxidant status (TOS), malondialdehyde (MDA) and fluorescent oxidation products (FOP) levels were determined.

TAS and TOS levels were measured by applying the TAS and TOS kits of Rel Assay Diagnostic to the Roche Cobas 6000 instrument (company, city, country?)

In the TAS measurement, antioxidants in the sample reduce dark blue-green coloured 2,2′-azino-bis (3-ethylbenzothiazoline-6-sulfonic acid) (ABTS) radical to colourless reduced ABTS form. The change of absorbance at 660 nm is related with the total antioxidant level of the sample. The assay is calibrated with a stable antioxidant standard solution, which is traditionally named as Trolox Equivalent, which is a vitamin E analogue.

In the TOS measurement, oxidants present in the sample oxidise the ferrous ion chelator complex to ferric ion. The oxidation reaction is prolonged by enhancer molecules, which are abundantly present in the reaction medium. The ferric ion makes a coloured complex with chromogen in an acidic medium. The colour intensity, which can be measured spectrophotometrically, is related to the total amount of oxidant molecules present in the sample. The assay is calibrated with hydrogen peroxide. TAS and TOS results are given in µmol/g protein.

MDA acts as a lipid peroxidation indicator and is a known tissue injury index. The levels of MDA were measured using the fluorometric method, in line with the description of Wasowicz et al. [9]. Following the reaction between thiobarbituric acid (TBA) and MDA, the reaction product was isolated in butanol, then a spectrofluorometric measurement was taken at a wavelength of 547 nm for emission and 525 nm for excitation. The standard was designated as a 0–5 µmol/L 1,1’,3,3’ tetraethoxypropane solution. Then, 50 µL homogenate was introduced into 10 ml glass tubes, each with 1 ml of distilled water in order to measure MDA levels in tissue. Following this step, 1 mL solution containing 29 mmol/L TBA was added to acetic acid and mixed. The samples were exposed to heat of between 95 °C and 100 °C for a period of 1 h using a water bath. After cooling the heated samples, they were mixed with 25 µL of 5 mol/L hydrochloric acid (HCL) and agitation was applied for a period of 5 min to extract the mixture of the reaction using 3.5 mL n-butanol. Following the separation of the butanol phase through centrifugation for 10 min at 1500 g, a fluorometer (HITACHI F-2500, Japan) was used to measure the fluorescence in the butanol extract at 547 nm and 525 nm wavelengths for emission and excitation, respectively. Solutions of 0–5 µmol/L 1,1’,3,3’ tetraethoxypropane were utilised as standard. The levels of MDA were shown as nmol/g protein.

Homogenised tissues were isolated using ethanol-ether (3/1, v/v) for FOP measurements, then these tissues were evaluated through a spectrofluorometer at wavelengths of 360 nm and 430 nm (wavelengths for excitation/emission) [10].

### 2.4. Histopathological evaluation

The liver tissue samples of each rat were preserved in storage containers including 10% formol. The tissue samples were sliced into strips at 3mm intervals and paraffin waxed into tissue cassettes for macroscopic investigation. The paraffin wax blocks were sliced into 4-micron sections and stained with H/E and Masson trichrome. The pathological evaluation was made by a single pathologist blinded to the groups. The samples were examined under a light microscope at × 40, × 100, × 200 and × 400 magnification. Histopathological changes were evaluated using the modified histological activity index scoring system (HAI). The HAI scoring system includes a total of five parameters as focal necrosis, portal inflammation, interphase hepatitis, confluent necrosis and fibrosis. Focal necrosis was graded as: none 0; Single focus at ×10 magnification - 1 (mild); 2–4 focus - 2 (moderate); 5–10 focus - 3 (severe), and >10 focus - 4 (extremely severe). Portal inflammation was graded as: none 0; mild and in some or all portal areas -  1 (mild); moderate and in some or all portal areas - 2 (moderate); moderately significant and in all portal areas - 3 (severe), and significant and in all portal areas - 4 (extremely severe). Interphase hepatitis was graded as: none 0; mild and focal or in some portal areas - 1 (mild); mild/moderate and focal or in most portal areas - 2 (moderate); moderate and in %50 > of portal areas or septum - 3 (severe), and severe and > %50 of portal areas or septum - 4 (extremely severe). Confluent necrosis was graded as: none (0/0); focal confluent necrosis - mild (1/1); zone 3 necrosis in some areas - moderate (2/2); zone 3 necrosis in most areas - moderate (2/3); zone 3 necrosis and also a few portal-central bridging necrosis - severe (3/4); zone 3 necrosis and also numerous portal-central bridging necrosis - severe (3/5), and panacinar or multiacinar necrosis - extremely severe (4/6). Fibrosis was graded as: none (0/0); fibrosis extension in some portal areas - mild (1/1); fibrosis extension in most of portal areas - mild (1/2); fibrosis extension in most of portal areas and also rare portoportal bridging - moderate (2/3); fibrosis extension in most of portal areas and also significant portoportal or portocentral bridging - moderate (2/4); significant portoportal or portocentral bridging and also rare nodules - severe (3/5), and probable or definite cirrhosis - extremely severe (4/6). 

Another parameter investigated beyond the HAI scoring was bile duct proliferation. In grading of bile duct proliferation, consecutive 5 areas in × 100 magnification were counted, and each proliferation was categorised as 0 (none), 1 (mild), 2 (moderate), and 3 (severe). Then, these numbers were averaged and categorised again similarly. Masson trichrome dye was used for the evaluation of fibrosis.

### 2.5. Statistical analysis

The data were analysed statistically using SPSS 22.0 software (IBM Corporation, Armonk, NY, USA). The Shapiro–Wilk’s test, skewness, and kurtosis values were used to analyse the data distribution. The data were presented as mean +/- SD or median (min-max) where applicable. The statistical analysis of the results was performed using unpaired Student’s t-tests and ANOVA models (with Tamhane’s post hoc test) with normally distributed data. For other types of data, the Mann–Whitney U test and the Kruskal–Wallis tests were used. The association between categorical variables was tested using the chi-square test or Fisher’s exact test. Significance was considered as P < 0.05.

## 3. Results

### 3.1. General

Throughout the study period, 2 rats from group 2 and 2 rats from group 3 died.

### 3.2. Oxidative stress parameters

Oxidative stress parameters are shown in Table 1 (TAS, TOS, MDA, and FOP). When TAS levels were compared between group 3 and group 2, the TAS levels in group 3 were found to be significantly higher than group 2 (P < 0.05). When group 3 and group 1 were compared, the TAS level was found to be significantly lower in group 3 (P < 0.05). When the TOS, MDA, and FOP levels were compared between group 3 and group 2, a statistically significant decrease was found (P < 0.05). When these parameters were compared between group 3 and group 1, they were found to be significantly higher (P < 0.05).

**Table 1:** Oxidative stress parameters of the groups.

Groups	TAS(mean ± SD)(µmol/g protein)	TOS(mean ± SD)(µmol/g protein)	MDA(mean ± SD)(nmol/g protein)	FOP(mean ± SD)(FP/g protein)
Group 1 (Sham)	204.43 ± 13.27a b	17.6 ± 3.99a d	9.17 ± 3.12a d	6.23 ±1.4a d
Group 2 (control)	157.85 ± 13.90a c	33.19 ± 4.04a e	24.61 ± 5.38a f	21.81 ±5.6a g
Group 3 (ABS)	180.74 ± 13.93b c	27.24 ± 3.54d e	18.43 ± 2.56d f	15.54 ±2.11d g
a) P < 0.001 for Group 1 versus Group 2b) P = 0.007 for Group 1 versus Group 3c) P = 0.016 for Group 2 and Group 3d) P < 0.001 for Group 1 and Group 3e) P = 0.022 for Group 2 versus Group 3f) P = 0.044 for Group 2 versus Group 3g) P = 0.047 for Group 2 versus Group 3				

Total antioxidant status (TAS), total oxidant status (TOS), malondialdehyde (MDA), fluorescent oxidant products (FOP), Ankaferd blood stopper (ABS)*P

### 3.3. Liver function tests 

The mean values of the liver function tests (ALB, AST, ALT, T.BIL, D.BIL) are summarised in Table 2. When group 2 and group 3 were compared, ALT levels were lower in group 3 but not to a statistically significant level (P = 0.691). Significant differences were determined irrespective of the ALB and AST values.

**Table 2:** Liver function parameters of the groups.

Groups	ALT (U/L)(mean ± SD)	AST (U/L)(mean ± SD)	Albumin(g/dl)(mean ± SD)	T Bilirubin(mg/dl)(mean ± SD)	D Bilirubin(mg/dl)(mean ± SD)
Group 1 (Sham)	66.50 ± 11.36a b	177.60 ± 29.44a b	3.80 ± 0.15a b	0.06 ± 0.01a b	0.03 ± 0.01a b
Group 2 (control)	412.13 ± 118.83a	825.25 ± 247.69a c	1.99 ± 0.16a d	7.46 ± 1.04a	7.46 ± 1.04a
Group 3 (ABS)	355.75 ± 100.63b	547.00 ± 107.44bc	2.83 ± 0.39bd	7.62 ± 1.4b	7.62 ± 1.44b
a) P < 0.001 for Group 1 versus Group 2b) P < 0.001 for Group 1 versus Group 3c) P = 0.048 for Group 2 and Group 3d) P = 0.001 for Group 2 versus Group 3					

Aminotransferase (AST),  alanine aminotransferase (ALT), standard deviation (SD), total (T), direct (D), Ankaferd blood stopper (ABS)

### 3.4. Histopathological analysis 

**Figure 1:**
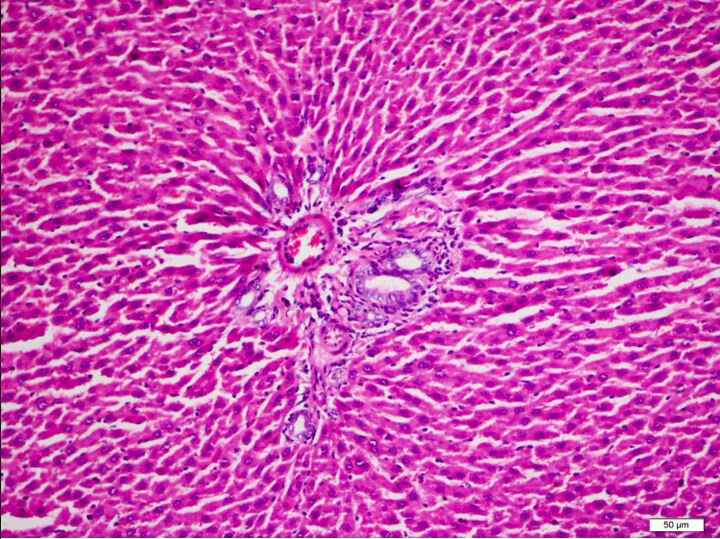
Group 1, liver microanatomy is preserved. Hepatocytes are observed to have radial extension around the portal area, and sinusoids appear normal. Portal inflammation, fibrosis and prominent bile duct proliferation are not present in the portal area. Hepatocytes appear normal and do not contain vacuolisation.

**Figure 2:**
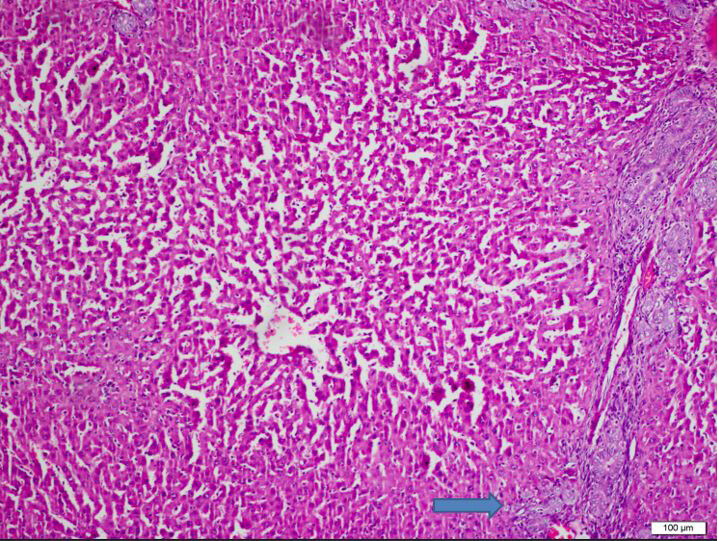
Group 2, the regular radial alignment of the hepatic cords seems to be largely lost. In addition to the prominent dilated appearance of sinusoids and central veins, severe bile duct proliferation and portal fibrosis and portal inflammation are present on the right side of the photo. There is focal interphase activity in the area marked with the arrow.

**Figure 3:**
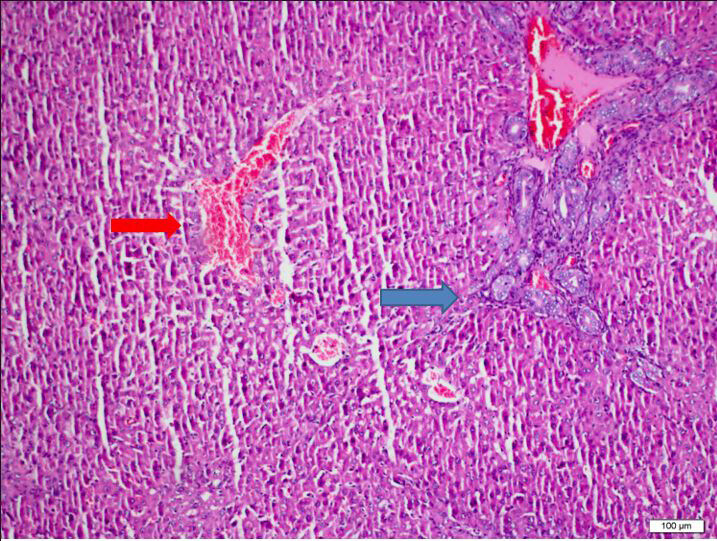
Group 2, focal interphase activity was observed in the blue star, zone 3 confluent necrosis around the central vein marked with arrow.

**Figure 4:**
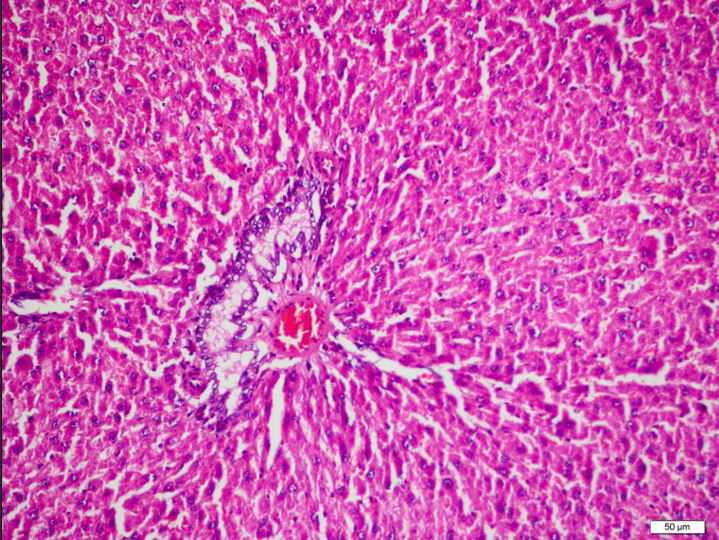
Group 3, hepatocytes show radial extension around the portal area and contain mild vacuolisation. Sinusoids appear normal. It does not contain congestion or significant inflammation. Portal inflammation, fibrosis and prominent bile duct proliferation are not present in the portal area.

Histopathological analyses according to the HAI scoring are given in Table 3. According to the statistical analysis of the HAI scores, statistically significant changes were found in all parameters between group 1 and group 2 (P < 0.05) (as shown in Figure 1, 2, and 3). Fibrosis, interphase activity and bile duct proliferation scores were statistically significantly lower in group 3 than in group 2 (P < 0.05) (as shown in Figure 4). Portal inflammation, focal inflammation and confluent necrosis scores were lower in group 3 than in group 2, but no statistically significant difference was observed.

**Table 3:** The histopathological scores of the groups.

Groups	Fibrosis median (min-max)	Portal inflammation median (min-max)	Focal inflammationmedian (min-max)	Confluent necrosis median (min-max)	Interface activity median (min-max)	Bile duct proliferation median (min-max)
Group 1 (Sham)	0 (0–1)a b	0 (0–1)d b	0 (0–1)	0 (0–1)e	0 (0–1)a	0 (0–1)a g
Group 2 (control)	3 (1–3)a c	2 (1–3)d	1 (0–2)	1 (0–2)e	2 (1–2)a f	4 (3–4)a h
Group 3 (ABS)	1 (1–2)a c	1 (1–2)b	0 (0–1)	0 (0–1)	1 (0–1)a f	1 (1–2)g h
a) P < 0.001 for Group 1 versus Group 2, b) P < 0.001 for Group 1 versus Group 3, c) P = 0.025 for Group 2 and Group 3, d) P = 0.001 for Group 1 and Group 2, e) P = 0.035 for Group 1 versus Group 2, f) P = 0.001 for Group 2 versus Group 3 , g) P = 0.001 for Group 1 versus Group 3, h) P < 0.001 for Group 2 versus Group 3						

Ankaferd blood stopper (ABS).

## 4. Discussion 

The most important finding in our study is that ABS exerts a hepatoprotective effect on rats that we have experimentally developed OJ. Both histopathological and biochemical results of this study showed better results in the OJ group given ABS compared to the control group in terms of liver function and tissue samples. Although ABS is known as a haemostatic agent, it may be an alternative treatment for liver damage due to the antioxidant and antiinflammatory components of OJ. Obstructive jaundice is a disease that occurs due to the blockage of the biliary intrahepatic or extrahepatic biliary tree. The inflammatory response to biliary obstruction results in characteristic changes in liver morphology in early and late phases [11].

Obstructive jaundice is an area of intensive experimental and clinical research. Bile duct ligation is the most comprehensive model used to produce obstructive jaundice in animals. Bile duct ligation causes an increase in oxidative stress markers in animals and also a decrease in antioxidant elements. Many recent studies have focused on the role of oxidative stress in hepatocellular damage during cholestasis [12–14].

Free oxygen radicals (FOR) caused by oxidative stress have an important role in the pathogenesis of certain diseases, including cancer. FOR is produced by parenchyma, inflammatory, and endothelial cells. Cytotoxic effects occur as levels increase, whereas when levels decrease, tissue damage decreases, and healing accelerates [15].

Biliary obstruction causes changes in bilirubin levels, bile salt metabolism and changes in enterohepatic circulation. These changes result in significant changes in liver functions [3].

Although the mechanism of damage due to bile salt accumulation is not fully understood, it is most likely due to inflammatory cell infiltration, accumulation of hydrophobic bile acids, endotoxemia, permeability of the mitochondrial membrane and toxic effects of FOR [16].

Reactive oxygen species are involved in necrosis and apoptosis of hepatocytes and contribute to hepatic stellate cell activation. Bile acids exacerbate oxidative damage by increasing FOR release from neutrophils and macrophages as well as increasing FOR production in mitochondria [17]. In the chemical analysis of ABS, many antioxidant molecules have been isolated (tocotrienols, members of the vitamin E family, tryptophan, estriol, galangine, apigenin, oenin, 3,4-diwanyltetrahydrofuran, tertiary butylhydroquinone (TBHQ), thymol, butylated hydroxyanisole (BHA), butylated hydroxytoluene (BHT), lycopene, enoxolone/glycyrrhetinic acid or glycyrrhetic acid and tomatine), and the concentrations of these have not been affected by exposure to synthetic gastric fluid. It has also been shown that ABS did not have a genetically modified organisms (GMO) process during the preparation process. In the same study, dioxin analyses in the ABS sample revealed that ABS does not contain toxic dioxin and chemical compounds like dioxin [18]. Apigenin, analysed in the content of ABS samples, is one of the main components responsible for bleeding and tissue degradation in the injured area. Apigenin is known to have inhibitory effects against snake venom metalloproteases. The predetermined antihemorrhagic activity and apigenin content of ABS will be investigated in future toxicology research [18,19]. In current studies, the antioxidant, tocotrienol, has also been found in ABS. As tocotrienol can activate apoptosis and suppress cellular proliferation and has antiangiogenic effects, it has been considered a potential anticancer agent [20–23]. Similarly, ABS can activate apoptosis, regulate cellular proliferation and suppress tumour angiogenesis. Experimental antineoplastic activities of ABS have been demonstrated in rats and cancer cell lines [24–29]. Since the desirable feature of an anticancer drug is that it can kill cancer cells and does not damage normal human cells, natural products are widely preferred in the production of anticancer drugs. ABS can significantly reduce the viability of cells and is reported to have no cytotoxic properties [30]. The antineoplastic effects of ABS were first investigated on the presence and development of Saos-2 osteosarcoma cells. That study showed a dose-dependent suppression of cell proliferation and a remarkable reduction in the survival of Saos-2 cells [31]. Göker et al. also investigated the effect of ABS on colon cancer cells (CaCo-2) and reported a decrease in cell viability [32]. Türk et al. showed growth inhibitory effects on primary melanoma cells and cell lines and that there were no cytotoxic effects on normal cells [33].

Resistance to chemotherapeutic drugs has become one of the current obstacles encountered in cancer treatment. In a study by Ghasemi et al., it was shown that by changing the genes in the oxidative phosphorylation pathway of ABS, some melanoma cell lines can be made more sensitive to etoposide [34]. It has also been reported that ABS may cause leukocyte infiltrations, vascularisation and fibroblast proliferation in the mucosal tissue, which may facilitate the wound healing process [35]. Kaya et al. used topical ABS on burn injury tissue and achieved a reduction in wound diameter and inflammation, accelerated tissue fibrosis, and wound contraction [36]. 

The antioxidant effects of quercetin, resveratrol and acetylcysteine were investigated in experimental jaundice in a study by Kawada et al. [37]. Padillo et al. performed an experimental cholestasis study and reported that melatonin reduced liver damage. In addition, bile duct obstruction has been shown to be associated with the depletion of many molecules and enzymes that possess antioxidant properties by triggering severe oxidative stress [16].

Ljunbuncic et al. investigated extrahepatic tissue damage in cholestatic liver disease and reported an association between experimental cholestasis and high lipid peroxidation in the brain, kidney and heart [38].

ABS is a topical haemostatic agent of plant origin that has been used in clinical practice in recent years. This herbal medicine consists of a standard mixture of Thymus vulgaris, Glycyrrhiza glabra, Vitis vinifera, Alpinia officinarum and Urtica dioica [5]. In addition to its haemostatic effects, ABS has antioxidant and antiinflammatory effects due to the components. For example, T. vulgaris has antioxidant effects such as the prevention of lipid peroxidation [39]. Glycyrrhiza glabra reduces the synthesis of lipopolysaccharide inflammatory mediators and inhibits reactive oxygen derivatives, prevents liposomal membranes, lipid peroxidation, and protects low-density lipoproteins from oxidation [40]. In the pathogenesis of gastric mucosal injury caused by acetyl salicylic acid (ASA) in rats, Hasgül et al. found that free oxygen radicals were formed and there was associated disruption of the antioxidant enzyme activity of cells and release of lipid peroxides. ABS has been shown to reduce oxidative and inflammatory changes caused by gastric mucosal damage caused by ASA in rat models [41].

In an experimental rat study carried out by Akbal et al., the gastrointestinal effect of oral high-dose ABS was investigated. No inflammation, fibrosis, biliary destruction or proliferation was observed in any of the groups in the histopathological examination of the liver [42].

To the best of our knowledge, this is the first study in literature to have examined the effect of ABS on experimental liver obstruction jaundice. Liver tissue samples were examined histopathologically and ABS was seen to significantly reduce inflammation and fibrosis. In addition, oxidative stress parameters and liver function tests were found to be decreased compared to the control group. Thus, the results of the study demonstrated the hepatoprotective effect of ABS on experimental obstructive jaundice. Nevertheless, further studies are needed to better determine its effectiveness in clinical practice.

## Statement of ethics

Animal experiments were performed in compliance with internationally accepted standards and have been approved by the appropriate institutional review body. Approval for this study was granted by the Animal Research Ethics Committee of Ankara Training and Research Hospital on 31.05.2018 with approval code 517.

## Author contributions

KK, AD, YU, TY, CE: Participated in research design, data analysis and interpretation. MS, IAK, EPE, SH: participated in experimental evaluations and histological analyses and monitored the animals. IAK, MS: Performed the biochemical evaluations. K.K: Wrote the paper. Finally, KK and MTB reviewed the article.

## Informed consent

Ethics committee approval was obtained from Ankara Training and Research Hospital Animal Experiments Local Ethics Committee (decision no. 46 on 31.05.2018).
